# Artificial Intelligence in Head and Neck Cancer: Innovations, Applications, and Future Directions

**DOI:** 10.3390/curroncol31090389

**Published:** 2024-09-06

**Authors:** Tuan D. Pham, Muy-Teck Teh, Domniki Chatzopoulou, Simon Holmes, Paul Coulthard

**Affiliations:** Barts and The London School of Medicine and Dentistry, Queen Mary University of London, Turner Street, London E1 2AD, UK; m.t.teh@qmul.ac.uk (M.-T.T.); d.chatzopoulou@qmul.ac.uk (D.C.); s.holmes@qmul.ac.uk (S.H.); p.coulthard@qmul.ac.uk (P.C.)

**Keywords:** artificial intelligence, head and neck cancer, oral cancer, imaging techniques, deep learning, natural language processing, early detection, personalized treatment, biomarker discovery, explainable machine intelligence

## Abstract

Artificial intelligence (AI) is revolutionizing head and neck cancer (HNC) care by providing innovative tools that enhance diagnostic accuracy and personalize treatment strategies. This review highlights the advancements in AI technologies, including deep learning and natural language processing, and their applications in HNC. The integration of AI with imaging techniques, genomics, and electronic health records is explored, emphasizing its role in early detection, biomarker discovery, and treatment planning. Despite noticeable progress, challenges such as data quality, algorithmic bias, and the need for interdisciplinary collaboration remain. Emerging innovations like explainable AI, AI-powered robotics, and real-time monitoring systems are poised to further advance the field. Addressing these challenges and fostering collaboration among AI experts, clinicians, and researchers is crucial for developing equitable and effective AI applications. The future of AI in HNC holds significant promise, offering potential breakthroughs in diagnostics, personalized therapies, and improved patient outcomes.

## 1. Introduction

### 1.1. Background on Head and Neck Cancer

Head and neck cancer (HNC) can develop in over 30 distinct regions within the head and neck [1,2]. These areas include the mouth and lips, where the oral cavity encompasses the lips, gums, and the inside of the cheeks. The voice box, or larynx, plays a crucial role in breathing, speaking, and swallowing, and it can also be affected by cancer. The throat, or pharynx, is divided into three sections: the nasopharynx (upper part of the throat behind the nose), the oropharynx (middle part of the throat), and the hypopharynx (bottom part of the throat), each of which can develop cancer. Salivary glands, which produce saliva to aid in digestion and keep the mouth moist, include the parotid, sublingual, and submandibular glands and can also be sites for cancer. The nasal cavity and paranasal sinuses, which are air-filled spaces around the nose and within the cheekbones and forehead, are potential sites for cancer development. Additionally, the area at the back of the nose and mouth, known as the nasopharynx, located behind the nose and above the back of the throat, is another region where cancer can occur. These cancers can vary in symptoms, treatment approaches, and prognoses, highlighting the importance of specialized care for each specific location within the head and neck.

Oral cancer, also referred to as mouth cancer or oral cavity cancer, is the most prevalent type of head and neck cancer (HNC) [1]. Head and neck squamous cell carcinomas, which develop from the mucosal lining in regions such as the oral cavity, pharynx, and larynx, are the most frequent malignancies occurring in the head and neck area [3]. The most prevalent type of oral cancer is squamous cell carcinoma (SCC), which originates from the squamous cells lining the oral cavity [4,5,6,7]. Although SCC is the most common, other less frequent forms include verrucous carcinoma [5], spindle cell squamous cell carcinoma [5], basaloid squamous cell carcinoma [5], and cancers of the salivary glands [8].

Globally, oral cancer poses a significant health challenge [9]. The World Health Organization’s 2013 report ranks oral diseases as the 13th most common cancer worldwide [10], while HNC types are among the top ten most prevalent cancers globally [11]. The high prevalence of oral cancer is particularly attributed to practices such as chewing tobacco and drinking alcohol [9]. Incidence rates vary across regions and demographics, with higher rates observed in males and older adults [12,13].

Several risk factors contribute to the development of oral cancer. Tobacco use, whether through smoking or smokeless products, is a major risk factor. Excessive alcohol consumption further increases the risk, especially when combined with tobacco use. Additionally, chewing betel quid and areca nut [14,15], common in parts of Asia, is strongly associated with oral cancer. Dietary factors [16], such as a low intake of fruits and vegetables, and a genetic predisposition due to family history of oral cancer can also play a role [17].

Oral cancer symptoms can be diverse, often leading to a delayed diagnosis as they may be mistaken for less serious conditions [18]. Common symptoms include persistent sores or ulcers in the mouth that do not heal, unexplained bleeding or pain, white or red patches on mucosal surfaces, and difficulty swallowing or speaking [19]. Additional signs, which are very common in non-malignant disease, include dental abscesses or a socket that fails to heal after a tooth extraction [20]. Other signs can be progressive limitation of mouth opening, pathological anesthesia due to sensory nerve involvement, fixation of tongue, and neuropathic tongue pain [21].

Diagnosis typically involves a thorough clinical examination, including inspection and palpation of the oral cavity and surrounding areas, mobility of teeth, mouth opening, sensory and motor disturbance, and tongue mobility. A biopsy is crucial for the histopathological examination of suspicious lesions. Imaging studies such as X-rays, computed tomography (CT), magnetic resonance imaging (MRI), and positron emission tomography (PET) scans are used to assess the extent of disease and aid in staging [19]. The staging of oral cancer is based on the TNM system, which evaluates tumor size, lymph node involvement, and metastasis to guide both prognosis and treatment options [22,23].

Treatment options for oral cancer depend on the stage and location of the disease. They may include surgery to remove the tumor and affected tissues, which can involve the removal of lymph nodes in the surrounding area. Radiotherapy is often used after surgery or as the primary treatment for localized tumors. Chemotherapy is typically reserved for advanced or metastatic cases and may be used in combination with radiation in a treatment approach known as chemoradiotherapy [19,24]. Emerging treatments such as targeted therapy and immunotherapy are also becoming increasingly important, focusing on specific cancer cell pathways or enhancing the body’s immune response to fight cancer [25,26].

The prognosis for oral cancer varies based on several factors including tumor stage, location, the patient’s overall health, and their response to treatment [22]. The most critical prognostic factors are lymph node involvement, tumor size, and the presence of distant metastases [22,27]. Generally, early-stage oral cancers have a better prognosis, but advanced stages and poorly differentiated tumors can lead to lower survival rates and increased morbidity.

Despite advances in treatment, challenges persist in the early detection, accurate diagnosis, and effective management of oral cancer [28]. Early diagnosis and recurrence prediction of oral cancer remain particularly problematic due to various factors [9,28,29], such as the following:

- Heterogeneity: oral cancer is not a single disease; it includes various subtypes with distinct genetic and clinical characteristics. This heterogeneity complicates diagnosis.
- Tissue sampling: accurate diagnosis often requires a biopsy of the affected tissue. However, obtaining a representative sample can be challenging, and false negatives may occur if the biopsy misses the cancerous area.
- Recurrence prediction: predicting the recurrence of oral cancer after treatment is challenging due to factors like tumor heterogeneity, incomplete removal of cancer cells during surgery, and resistance to therapy.
- Imaging limitations: while medical imaging, such as CT scans, is valuable for cancer diagnosis and staging, it may not always detect small or early-stage lesions accurately. This can lead to underdiagnosis.
- Patient variability: patient-related factors, such as lifestyle choices, overall health, and genetic predisposition, can influence both the initial diagnosis and the likelihood of recurrence.
- Post-treatment changes: treatments for oral cancer, such as surgery and radiation therapy, can result in changes to the oral cavity, affecting speech, swallowing, and quality of life. Distinguishing between post-treatment changes and cancer recurrence can be difficult.
- Limited biomarkers: currently, there are limited specific biomarkers for oral cancer, making it challenging to identify individuals at high risk.
- Patient awareness and access: some patients may lack awareness of oral cancer risk factors and symptoms, and others may face barriers to accessing healthcare, delaying diagnosis.

**Figure 1:**
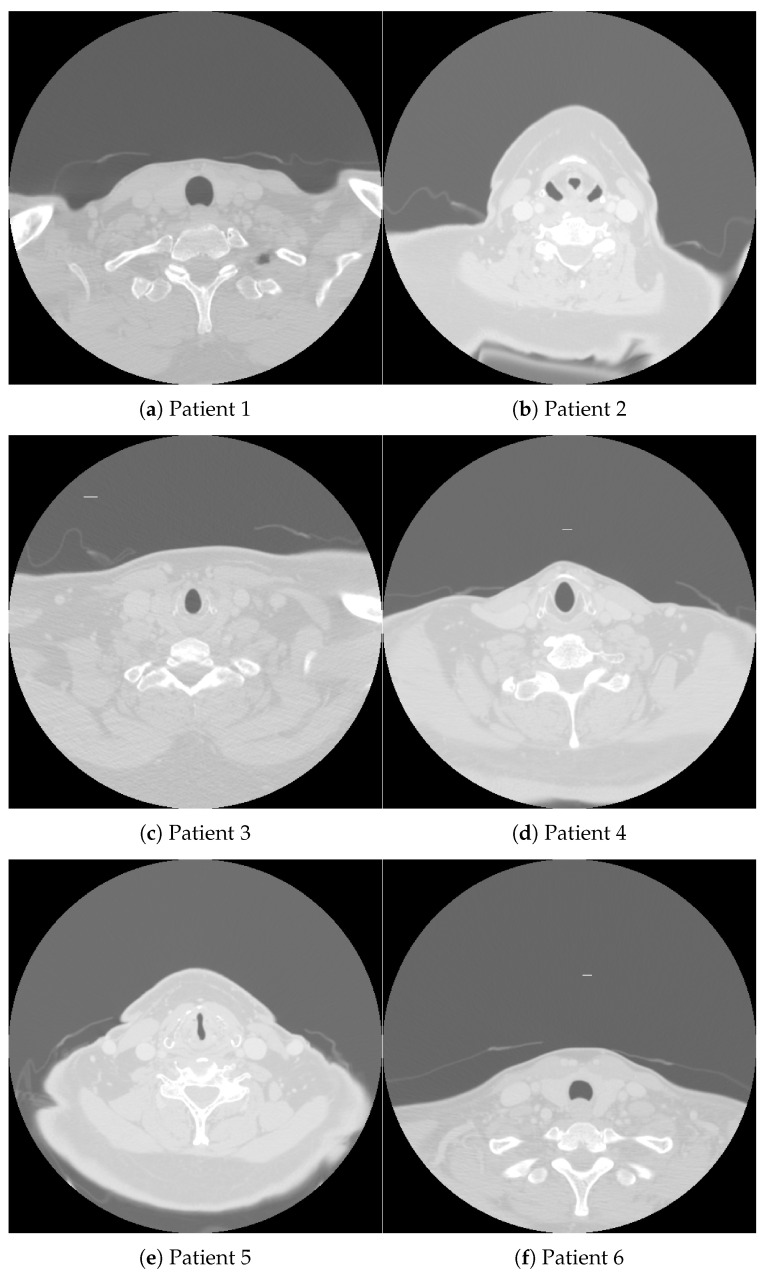
CT images obtained from oropharynx cancer patients whose demographic and clinical data are detailed in Table 1.

Figure 1, accompanied by Table 1, shows CT images of a cross-sectional view of the upper chest and lower neck region from four patients diagnosed with oropharynx cancer (OPC). These images were sourced from a publicly available database [30], which supports the development of artificial intelligence (AI) methods aimed at identifying OPC patients with varying risks of disease recurrence after definitive radiotherapy. Specifically, the database aids in distinguishing patients with the lowest likelihood of recurrence from those at risk of local recurrence in the primary tumor site.

**Figure 2:**
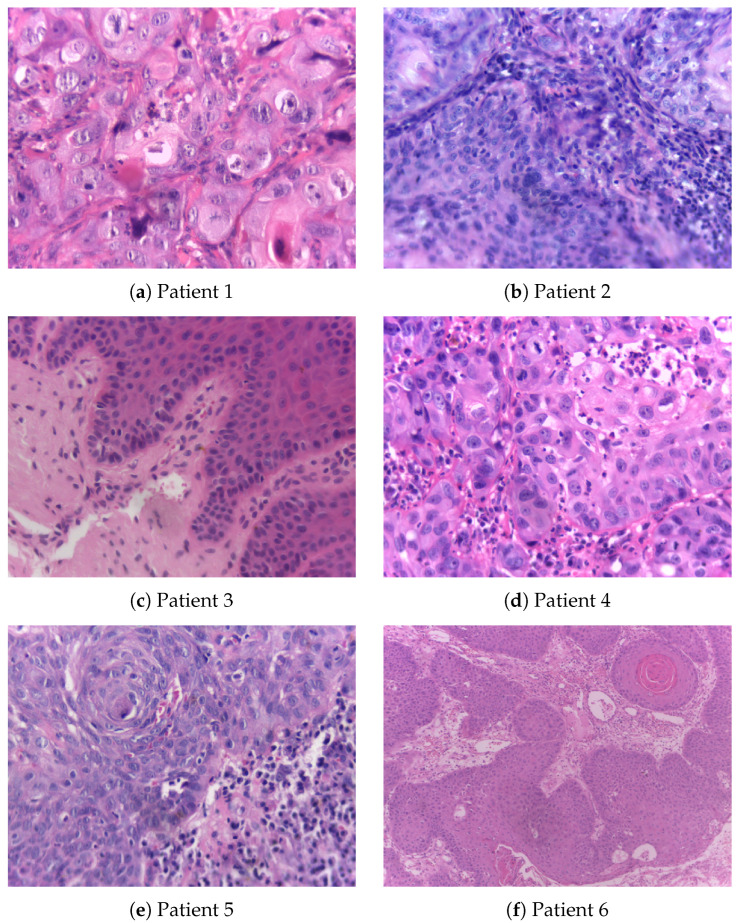
Histopathological images, resized to 0.3 × original images, obtained from patients with oral squamous cell carcinoma and leukoplakia whose demographic and clinical data are detailed in Table 2.

Figure 2, accompanied by Table 2, shows histopathological images of oral squamous cell carcinoma (OSCC) and leukoplakia sourced from the publicly accessible NDB-UFES database [31]. This database is intended to support AI researchers in their efforts to develop advanced diagnostic tools that can assist clinicians and pathologists in the automated and accurate diagnosis of potentially malignant oral disorders and OSCC.

Ongoing research aims to address challenges in HNC by developing more precise diagnostic tools, innovative treatment modalities, and preventive strategies. The integration of AI holds promise for improving early detection and personalizing treatment approaches, potentially transforming the landscape of HNC care [32,33,34,35].

### 1.2. Review Objectives

The primary objective of this review is to explore and elucidate the role of AI in the field of HNC, focusing on its applications, advancements, and impact on clinical practice. HNC presents significant challenges in early detection, accurate diagnosis, and effective treatment. Integrating AI into these areas offers potential solutions that could significantly enhance current approaches. This review aims to provide a comprehensive overview of how various AI technologies are being utilized to improve the management of HNC.

Another key goal is to evaluate the current state of AI applications in diagnosis, prognosis, and prediction for HNC. By examining recent advancements in AI-driven diagnostic tools, such as automated image analysis, the review aims to highlight how AI improves early detection rates and diagnostic accuracy. Additionally, it will assess how AI models contribute to prognostic tools that forecast patient outcomes and to predictive models that anticipate specific responses to treatments, thereby supporting personalized treatment strategies.

The review also aims to identify and address the challenges and limitations associated with integrating AI into HNC care. This includes exploring issues related to data quality, algorithmic bias, and the practical implementation of AI technologies in clinical settings. By shedding light on these challenges, the review seeks to provide insights into the barriers that need to be overcome to fully leverage the benefits of AI in this field.

Furthermore, the review will explore future directions for AI in HNC research and clinical practice. This involves discussing emerging trends, potential innovations, and the interdisciplinary collaborations required to advance AI technologies. By considering these future prospects, the review seeks to outline a roadmap for how AI can continue to evolve and contribute to improved outcomes in the management of HNC.

While the review primarily focuses on oral cancer, which is the most common type of HNC, “head and neck cancer” is included in the title to reflect the inclusion of other HNC subtypes. This choice underscores the broader relevance of the findings and discussions to the spectrum of HNC. Additionally, several themes explored in the manuscript—such as diagnostic techniques, treatment innovations, and the role of artificial intelligence—are applicable across various HNC subtypes. Highlighting “head and neck cancer” in the title ensures that readers researching or treating other forms of HNC, beyond just oral cancer, can benefit from the insights provided. This approach helps the manuscript reach a wider audience within the field and facilitates cross-disciplinary learning that could contribute to advancements across different HNC subtypes.

### 1.3. Selection Criteria

References for this review were selected based on stringent criteria, focusing primarily on papers published within the past five years. Priority was given to studies featured in reputable journals indexed by major databases such as the National Library of Medicine, Elsevier Databases, IEEE Xplore, and Web of Science. Additionally, the selection emphasized high-quality journals and those with rigorous peer-review processes to ensure the inclusion of the most current and relevant research in the field.

## 2. AI Technologies and Methodologies

### 2.1. Machine Learning Techniques

Machine learning represents a pivotal branch of AI that enables systems to learn from and make predictions based on data. This capability is central to many applications in healthcare, including the management of HNC. The fundamental techniques within machine learning include supervised learning, unsupervised learning, and reinforcement learning, each of which offers unique approaches to data analysis and decision-making.

Supervised learning is a technique where algorithms are trained on labeled datasets, meaning that each training example is paired with an outcome or target value. The primary goal of supervised learning is to build a model that can predict the outcome for new, unseen data based on the patterns learned from the training set. In the context of HNC, supervised learning algorithms can be employed to analyze medical images, such as histopathological slides or radiographs, to identify features indicative of malignancy [36,37,38]. By learning from examples of both benign and malignant cases, these models can assist in diagnosing HNC with increased accuracy. Common algorithms used in supervised learning include decision trees, support vector machines, and artificial neural networks (ANNs).

Unsupervised learning, in contrast, involves training algorithms on datasets without predefined labels or outcomes. Instead of predicting a specific result, unsupervised learning aims to uncover hidden patterns or structures within the data. This technique is particularly useful for exploratory data analysis and feature extraction. For HNC research, unsupervised learning can be applied to cluster patient data based on similarities in genetic profiles or imaging features, potentially revealing new subtypes of the disease or novel biomarkers [38,39,40]. Techniques such as clustering algorithms and principal component analysis are commonly used in unsupervised learning to organize and interpret complex datasets.

Reinforcement learning represents a different approach, focusing on training algorithms to make sequences of decisions by interacting with an environment. In this paradigm, the algorithm learns to achieve a goal through trial and error, receiving rewards or penalties based on its actions. Reinforcement learning is well-suited for dynamic and complex decision-making scenarios, such as optimizing treatment plans for HNC patients [41,42]. By simulating various treatment strategies and their outcomes, reinforcement learning algorithms can help identify the most effective approaches for individual patients [43]. This technique can also be used to refine surgical procedures or enhance patient monitoring strategies [44,45].

Each of these machine learning techniques offers distinct advantages and can be applied to various aspects of HNC research and treatment. Supervised learning excels in predictive modeling with labeled data, unsupervised learning provides insights into complex and unlabeled datasets, and reinforcement learning supports adaptive decision-making in dynamic environments. Together, these techniques contribute to the development of advanced tools and strategies for improving the management of HNC.

### 2.2. Deep Learning Approaches

Deep learning [46] is a specialized branch of machine learning that utilizes ANNs with multiple layers, known as deep neural networks, to model and analyze complex patterns in data. This approach has gained prominence due to its remarkable ability to process large volumes of data and uncover intricate relationships, making it particularly useful in the field of healthcare and, specifically, in HNC management.

One of the most widely used deep learning techniques is the convolutional neural network (CNN), which is especially effective in image analysis tasks. CNNs are designed to automatically and adaptively learn spatial hierarchies of features from input images through convolutional layers. In the context of HNC, CNNs can be employed to analyze medical images, such as histopathological slides, radiographs, and intraoral photographs [47,48,49,50,51]. By training on large datasets of labeled images, CNNs can learn to identify and classify features indicative of cancerous lesions with high accuracy. This capability enhances the precision of diagnostics and aids in the early detection of HNC, potentially improving patient outcomes.

Recurrent neural networks (RNNs) are another deep learning technique that is particularly suited for sequential data analysis. RNNs possess the ability to retain information from previous inputs in the sequence, making them ideal for tasks that involve temporal dependencies. In HNC research, RNNs can be used to analyze time-series data, such as patient health records or treatment response patterns over time [49,52,53]. This allows for the modeling of disease progression and the prediction of future health events, facilitating personalized treatment planning and monitoring.

Autoencoders represent a different type of deep learning architecture used for unsupervised learning [49,54]. These neural networks are designed to encode input data into a reduced-dimensional representation and then decode it back to the original data. The process of learning efficient data representations can be leveraged in HNC research for tasks such as anomaly detection, where the goal is to identify unusual patterns or outliers in medical images or genetic data [55]. Autoencoders can help in discovering subtle features and subtypes that may be indicative of early-stage cancer or other significant abnormalities [56].

Generative adversarial networks (GANs) have also emerged as a powerful deep learning technique. GANs consist of two neural networks, a generator and a discriminator, that compete against each other. The generator creates synthetic data samples, while the discriminator evaluates their authenticity. This adversarial process leads to the generation of highly realistic data, which can be useful in augmenting training datasets for HNC diagnostics. For example, GANs can generate synthetic medical images to train other deep learning models, improving their robustness and performance even when limited real-world data are available [49,57].

Another recent advancement in deep learning for medical image classification is the vision transformer (ViT) network. Vision transformers have revolutionized image processing by leveraging the transformer architecture, initially developed for natural language processing. ViTs break down an image into a sequence of patches, treating each patch similarly to a word in a sentence. This approach allows the model to capture long-range dependencies and relationships across the entire image. In the context of HNC, ViTs can be employed to classify medical images with a high degree of accuracy. They excel in identifying subtle patterns and variations in medical imagery, potentially leading to more precise diagnostics. ViTs have demonstrated state-of-the-art performance in various image classification tasks, offering a powerful tool for enhancing the analysis of complex medical images in HNC research [49,58].

### 2.3. Natural Language Processing

Natural language processing (NLP) is a crucial area within AI that focuses on the interaction between computers and human language. NLP enables machines to understand, interpret, and generate human language in a way that is both meaningful and contextually relevant. This technology has significant applications in healthcare, particularly in literature mining and patient data analysis [59,60,61,62], which are essential for advancing research and improving clinical outcomes in HNC.

In literature mining, NLP techniques are used to extract valuable information from vast amounts of unstructured textual data found in scientific publications, research articles, clinical reports, and other medical literature. The exponential growth of scientific knowledge makes it challenging for researchers and clinicians to stay up-to-date with the latest developments. NLP can automate the process of identifying relevant studies, summarizing findings, and extracting critical information about HNC, such as risk factors, genetic markers, treatment outcomes, and emerging therapies. By employing NLP algorithms, researchers can efficiently sift through large datasets, uncovering insights that might be missed through manual review. This capability enhances the ability to conduct comprehensive literature reviews and meta-analyses, facilitating evidence-based decision-making and guiding future research directions.

In patient data analysis, NLP plays a vital role in extracting and analyzing information from electronic health records (EHRs), clinical notes, pathology reports, and other healthcare documentation [63,64,65]. These records often contain rich, detailed narratives about a patient’s medical history, symptoms, diagnoses, treatments, and outcomes. NLP can transform this unstructured text into structured data, making it easier to analyze and integrate with other clinical information. For example, NLP algorithms can identify mentions of specific symptoms, track disease progression, and correlate treatment responses with patient outcomes. In the context of HNC, this can lead to more accurate diagnoses, better monitoring of disease recurrence, and the identification of patterns that inform personalized treatment plans [32,66].

Furthermore, NLP can facilitate the development of clinical decision support systems (CDSSs) by providing real-time analyses of patient data and generating recommendations based on the latest clinical guidelines and research findings [67,68,69]. These systems can alert clinicians to potential issues, suggest diagnostic tests, and recommend treatment options, all of which contribute to improved patient care. For HNC patients, NLP-driven CDSSs can assist in identifying high-risk individuals, suggesting early interventions, optimizing treatment strategies based on individual patient profiles, and attaining fairness in dental care [70].

## 3. Diagnostic Applications

### 3.1. Early Detection

Early detection and screening are critical components in the management of HNC, significantly improving the chances of successful treatment and survival. AI plays a transformative role in these areas by enhancing the analysis of medical images and automating the detection and classification of lesions, leading to more accurate and timely diagnoses. For example, a study [71] examined 1636 photographic images of lesions from 152 oral cancer patients, covering conditions such as squamous cell carcinoma, pleomorphic adenoma, lipoma, and others. Among various AI models tested, the ResNet architecture with 152 layers achieved the highest classification accuracy at 81%. One of the most recent reviews [72] evaluated techniques for early oral cancer detection, highlighting the effectiveness of histopathology images and various image processing steps like enhancement and classification. It compared multiple AI approaches—including deep learning, conventional machine learning, data mining, genetic algorithms, and fuzzy computing—finding that deep learning algorithms provided the highest detection accuracy, exceeding 90%.

In the analysis of histopathological slides and oral cavity photographs, AI employs advanced techniques like deep learning to identify patterns and abnormalities with high precision. Histopathological data include normal, benign, malignant, and potentially malignant oral disorders of the mucosa [33]. Deep learning models, particularly CNNs, can process and interpret complex visual data, detecting minute changes that might indicate the presence of cancerous cells. These models are trained on large datasets of labeled images, enabling them to distinguish between malignant and benign tissues. This capability is vital in histopathology, where early cancer signs can be subtle and easily overlooked [73]. Additionally, the data encompass normal and malignant tissues, as well as the expression of VEGF-C, VEGF-D, NRP1, NRP2, CCR7, and SEMA3E in immunohistochemistry images from patients with OSCC, cervical lymph node metastasis from primary tongue tumors, head and neck squamous cell carcinoma, tumor-associated stroma, infiltrating lymphocytes, and oral submucosal fibrosis. AI not only enhances diagnostic accuracy but also reduces the time needed to analyze slides, allowing pathologists to focus on cases that require more detailed examination.

High-resolution photographs taken during routine dental check-ups or specialized imaging sessions can be examined by AI algorithms to identify potential lesions. These systems can detect abnormalities such as leukoplakia, erythroplakia, and other precancerous conditions with a high degree of sensitivity [74,75]. For instance, autofluorescence and white light images of the buccal mucosa, gingiva, soft palate, vestibule, floor of the mouth, and tongue were utilized to classify oral cancer [76]. Images were categorized into normal and suspicious, with the latter group including both oral potentially malignant lesions and malignant lesions. Several transfer learning classifiers were applied to this task, with the VGG-CNN-M model [77] delivering the best performance, achieving an accuracy of 87%, sensitivity of 85%, and specificity of 89%. Early identification of such lesions allows for timely intervention, potentially preventing the progression to invasive cancer. AI-powered tools can be integrated into standard dental equipment, providing real-time analysis and alerts during examinations, thereby supporting dentists in making informed clinical decisions.

Exfoliative cytology [78,79,80] involves collecting cells from the surface of the oral mucosa, which are then examined microscopically to detect any abnormalities. In the context of the oral cavity, AI can significantly enhance the analysis of exfoliative cytology samples by automating the identification of nuclear parameters [81] and cellular changes that might indicate precancerous conditions like leukoplakia [74]. By utilizing smart cytology with remote diagnosis demonstrated a significant improvement in distinguishing between OSCC and high-grade dysplasia versus low-grade dysplasia, achieving an accuracy of 60% with manual professional assessment compared to 90% using an ANN-based risk stratification model [82].

AI algorithms, particularly those based on deep learning, can analyze large datasets of cytology images to identify subtle differences in cellular morphology and arrangement that may be difficult for human observers to detect [83]. For instance, in patients with a history of smoking, AI can help differentiate the cytological features of oral epithelial dysplasia [84] by recognizing specific cellular alterations commonly associated with tobacco exposure, such as increased nuclear-to-cytoplasmic ratio, hyperchromasia (darkly staining nuclei), and irregular nuclear borders. These changes are more pronounced in smokers compared to non-smokers, who might show milder or different cellular alterations in leukoplakic lesions. The ability of AI to compare and analyze these differences at a granular level will enable a more accurate and early detection of potentially malignant transformations in leukoplakia among smokers, thereby providing critical information for personalized patient management and targeted interventions.

AI systems can be designed to automatically scan images for lesions, classify them based on their characteristics, and assess their likelihood of being malignant [72,74]. This automation is particularly valuable in high-throughput screening scenarios, where large numbers of images need to be evaluated quickly. AI algorithms can sort through these images, highlighting those that require further review by a specialist. This not only streamlines the workflow in medical and dental practices but also ensures that no potential cases are overlooked due to human error or fatigue. For instance, mobile-phone images captured using the Mobile Mouth Screening Anywhere (MeMoSA) app (https://memosa.my) were utilized for the classification of oral lesions to support the early detection of oral cancer [85]. These images included normal, benign, oral potentially malignant disorders (both high and low risk), and malignant lesions. The ResNet-101 deep learning model was employed for this classification task. The model achieved an F1 score of 87%, precision of 85%, and recall of 90% in identifying images containing lesions, and an F1 score of 78.3%, precision of 67%, and recall of 94% in identifying images that required referral.

Moreover, AI can integrate data from multiple sources to improve the accuracy of lesion detection and classification. For instance, combining image analysis with patient history, genetic information, and other diagnostic data can enhance the overall assessment and provide a more comprehensive understanding of the patient’s condition [72,86]. In the study conducted by de Lima et al. [86], the authors examined the significance of incorporating clinical and demographic data alongside histopathological image analysis for computer-aided diagnosis of oral leukoplakia and carcinoma. The clinical and demographic data included lesion location, size, patient gender, age group, alcohol and cigarette use, and sun exposure habits. The research utilized deep learning models to classify samples as OSCC, leukoplakia with dysplasia, and leukoplakia without dysplasia. When combining images with clinical and demographic data, the best balanced classification accuracy (BCC) reached 95%, compared to 93% using images alone, for differentiating between leukoplakia and OSCC. For distinguishing between samples with or without dysplasia, the best BCC improved to 88% with the combined approach versus 83% using only images. Additionally, when differentiating OSCC from leukoplakia, with or without dysplasia, the best BCC was 83% with combined data compared to 64% with just imaging data. This demonstrates the value of integrating demographic and clinical information into AI-based diagnostic systems.

### 3.2. Biomarker Discovery

AI is playing an increasingly important role in the discovery of biomarkers for HNC. Biomarkers are biological molecules that can be used to detect the presence of disease, predict disease progression, and monitor treatment response. The identification of reliable biomarkers is essential for developing diagnostic tools, prognostic models, and personalized treatment plans.

The ability of AI to handle and analyze vast amounts of biological data makes it particularly well-suited for biomarker discovery [87,88]. Machine learning algorithms can sift through complex datasets to identify patterns and correlations that may indicate the presence of biomarkers [89,90]. Datasets often include radiomic, genomic, proteomic, and metabolomic information, each containing millions of data points that are beyond the capacity of traditional analytical methods [37,91].

Salivary biomarkers have the potential to enhance the accuracy, speed, and effectiveness of diagnosing and monitoring oral and maxillofacial diseases. These biomarkers have been applied to conditions such as periodontal diseases, dental caries, oral cancer, temporomandibular joint dysfunction, and salivary gland diseases. However, due to the variable accuracy of salivary biomarkers during validation, employing modern analytical methods for selecting and implementing biomarkers from the extensive multi-omics data available could improve their performance. Artificial intelligence is one such advanced technique that could optimize the use of salivary biomarkers for the diagnosis and management of oral and maxillofacial diseases [92].

In genomics, AI can analyze DNA sequences to identify mutations, gene expression profiles, and epigenetic changes associated with HNC [93]. Machine learning models can be trained to distinguish between normal and cancerous tissues based on their genetic signatures. By integrating data from various genomic studies, AI can identify potential genetic biomarkers that could serve as targets for early detection or therapeutic intervention.

Proteomics, the large-scale study of proteins, is another area where AI contributes to biomarker discovery. AI algorithms can analyze protein patterns of alteration, expression levels, and modifications to identify those that are uniquely associated with HNC [94,95]. This involves processing complex mass spectrometry data to detect subtle changes in protein profiles. Identifying such protein biomarkers can lead to the development of blood tests or other assays that detect HNC at an early stage.

## 4. Prognostic and Predictive Models

### 4.1. Risk Assessment Tools

AI has emerged as a powerful tool in the development of risk assessment models for predicting disease progression and patient outcomes in HNC [32,96,97,98]. These AI-driven models utilize vast amounts of clinical, demographic, and molecular data to provide more accurate and individualized risk predictions. This capability is transforming how clinicians assess patient prognosis and make treatment decisions.

AI models for predicting disease progression in HNC typically incorporate machine learning algorithms trained on historical patient data. These datasets include a wide array of variables such as patient age, tumor characteristics, genetic information, treatment history, and lifestyle factors like smoking and alcohol use. By analyzing these factors, AI models can identify patterns and risk factors associated with more aggressive disease behavior and poorer outcomes. For example, machine learning algorithms can detect subtle interactions between genetic mutations and clinical features that may indicate a higher likelihood of metastasis or recurrence [40,99,100].

One of the significant advantages of AI in risk assessment is its ability to handle and integrate diverse data types, leading to more comprehensive risk profiles. Traditional statistical methods often struggle with the complexity and high dimensionality of such data, whereas AI and advanced data-science algorithms excel in extracting meaningful insights [89,101]. This allows for the creation of multi-factorial risk models that consider the interplay between various predictors, resulting in more robust and personalized risk assessments.

AI models also improve the accuracy of survival predictions for HNC patients [32,102,103,104,105,106,107,108]. By analyzing large datasets, these models can generate survival curves and estimate individual patient survival probabilities based on their specific characteristics. This information is invaluable for clinicians and patients when discussing prognosis and planning treatment strategies. For instance, an AI model might predict that a patient with certain genetic markers and tumor features has a higher risk of recurrence, prompting more aggressive monitoring and follow-up care [109,110,111].

Furthermore, AI-driven risk assessment tools can be continuously updated and refined as new data become available. This dynamic nature ensures that the models remain current and reflect the latest understanding of disease biology and treatment outcomes. As more data are collected, the predictive accuracy of these models can improve, providing increasingly precise risk estimates. In addition to predicting disease progression, AI models can also forecast patient responses to various treatments. By analyzing historical treatment data and outcomes, AI can help identify which patients are most likely to benefit from specific therapies. This predictive capability supports the development of personalized treatment plans, optimizing therapeutic efficacy while minimizing unnecessary side effects. For example, an AI model might indicate that a patient with a particular genetic profile is more likely to respond to a targeted therapy, guiding clinicians toward more effective treatment choices.

### 4.2. Personalized Treatment Planning

AI is playing a transformative role in personalized treatment planning for HNC by tailoring treatment regimens based on individual patient data [112,113]. This approach leverages the ability of AI to analyze complex datasets and identify patterns that can inform more precise and effective treatment strategies, thereby improving patient outcomes and minimizing adverse effects.

One of the primary advantages of AI in personalized treatment planning is its capacity to integrate and analyze diverse types of data. Patient information such as genetic profiles, tumor characteristics, medical history, lifestyle factors, and treatment responses can be comprehensively evaluated by AI algorithms. This holistic analysis enables the identification of unique patient subgroups and the prediction of how different individuals will respond to various treatments. For instance, genetic data can reveal specific mutations or biomarkers that indicate susceptibility to certain drugs, allowing for the selection of targeted therapies that are more likely to be effective for a particular patient [114,115,116].

AI algorithms, particularly machine learning models, can process this vast amount of data to develop predictive models that guide treatment decisions. These models can analyze historical treatment outcomes to determine which therapies have been most successful for patients with similar profiles. For example, AI has been utilized to forecast immunotherapy responses by analyzing immune signatures, medical imaging, and histological data. These characteristics could also be extremely beneficial in managing cancer immunotherapy due to their continually improving ability to enhance diagnostic precision, optimize treatment planning, predict care outcomes, and lower human resource expenses [117].

Moreover, AI facilitates the real-time adaptation of treatment plans based on ongoing patient responses. Continuous monitoring of patient data, such as changes in tumor size, biomarkers, and side effects, allows AI systems to adjust treatment regimens dynamically. If a patient’s tumor is not responding to the initial treatment, AI can analyze the data and suggest alternative therapies that might be more effective [57,118]. This adaptive approach ensures that patients receive the most appropriate care at each stage of their treatment journey, improving the likelihood of successful outcomes.

In radiation therapy, AI can optimize treatment planning by precisely targeting cancerous tissues while sparing healthy surrounding tissues. Advanced AI techniques can analyze imaging data to delineate tumor boundaries accurately and calculate the optimal radiation dose distribution [119,120]. This precision reduces the risk of radiation-induced side effects and enhances the overall effectiveness of the treatment. Additionally, AI can predict potential complications based on patient-specific factors and suggest adjustments to the radiation plan to mitigate these risks.

The integration of AI in personalized treatment planning also extends to HNC surgical interventions [121,122]. AI-driven analyses of preoperative imaging and patient data can assist surgeons in planning the most effective surgical approach, ensuring complete removal of cancerous tissues while preserving vital structures. Postoperative outcomes and potential complications can also be predicted using AI, guiding postoperative care and monitoring to enhance recovery and reduce the risk of recurrence [123,124].

The role of AI in personalized treatment planning is not limited to the selection and optimization of therapeutic interventions. It also encompasses patient education and community engagement [47,75,125]. AI-driven platforms can provide patients with tailored information about their treatment options, potential outcomes, and side effects, helping them make informed decisions about their care. This personalized communication enhances patient satisfaction and adherence to treatment plans, contributing to better overall outcomes.

The application of AI in personalized treatment planning for cancer represents a significant advancement in oncology care [126,127]. By integrating and analyzing individual patient data, AI enables the development of tailored treatment regimens that maximize efficacy and minimize adverse effects. This personalized approach enhances the precision of HNC care, supports dynamic adaptation of treatment plans, and improves patient engagement and satisfaction. As AI technologies continue to evolve and become more integrated into clinical practice, their impact on personalized treatment planning and patient outcomes in HNC is expected to grow, leading to more effective and efficient healthcare delivery.

### 4.3. Monitoring and Surveillance

AI is significantly enhancing the monitoring and surveillance of HNC by improving the tracking of disease recurrence [128,129,130]. These AI-driven advancements facilitate timely interventions and more effective management of patient health, ultimately improving outcomes and quality of life.

The capabilities of AI in data analysis and pattern recognition make it an invaluable tool for tracking disease recurrence in HNC patients. By continuously analyzing patient data from various sources, such as electronic health records, imaging studies, and laboratory results, AI can identify early signs of recurrence that might be missed during routine follow-ups. Machine learning models can be trained to recognize specific patterns and markers associated with cancer relapse, enabling clinicians to detect and address these issues promptly. For example, changes in imaging data [37,99], subtle shifts in biomarker levels [131], or new symptoms reported in patient records [132,133] can all signal a potential recurrence, prompting further investigation and early intervention.

Furthermore, AI-powered surveillance systems can integrate data from wearable devices [134] and mobile health applications [135] to provide continuous monitoring of patients’ health status. These technologies can track vital signs, physical activity, and other health metrics in real time, alerting healthcare providers to any abnormalities that may indicate a recurrence or other health issues. This continuous monitoring allows for a proactive approach to patient care, ensuring that any signs of recurrence are detected early, when they are most treatable.

In addition to tracking disease recurrence, AI plays a crucial role in monitoring patient compliance with treatment regimens [136,137,138]. Non-compliance with prescribed treatments can significantly impact the effectiveness of cancer therapy and increase the risk of recurrence. AI systems can analyze data from various sources, such as pharmacy records, appointment attendance, and self-reported information from patients, to assess adherence to treatment protocols. Machine learning algorithms can identify patterns of non-compliance and predict which patients are at higher risk of deviating from their treatment plans.

To address issues of non-compliance, AI can provide personalized interventions and support [74,139]. For example, the use of a mobile app allows for detailed, real-time tracking of patients receiving treatment with oral antineoplastic agents [140]. The algorithm’s automatic suggestions enhanced the efficiency of healthcare resources, aided in the early identification of adverse events, empowering patients to enhance the safety of their treatment. These applications can also offer educational resources and motivational support to encourage patients to stay on track with their treatment plans. Additionally, AI can facilitate virtual check-ins and telehealth consultations [141,142], making it easier for patients to access care and communicate with their healthcare providers, thereby improving adherence and overall engagement with their treatment.

## 5. Therapeutic Applications

### 5.1. Drug Discovery

AI is revolutionizing drug discovery, particularly in the field of cancer, by identifying new therapeutic targets and drug compounds with remarkable efficiency and precision [143,144,145,146,147,148]. Traditional drug discovery processes are often time-consuming and costly, involving extensive trial-and-error experimentation. In contrast, AI accelerates this process by leveraging vast amounts of biomedical data and sophisticated algorithms to uncover novel insights and promising leads.

One of the primary ways AI contributes to drug discovery is through the identification of new therapeutic targets. AI algorithms can analyze large datasets from genomics, proteomics, and other omics fields to identify molecular pathways and genetic mutations associated with HNC. By recognizing patterns and relationships within these datasets, AI can pinpoint specific proteins, genes, or pathways that play critical roles in cancer development and progression. For example, machine learning models can sift through genomic data to identify mutations or variants that drive tumor growth, offering potential targets for new drugs [149,150]. This capability allows researchers to focus their efforts on the most promising targets, streamlining the initial stages of drug discovery.

In addition to target identification, AI significantly enhances the process of discovering new drug compounds. Traditional methods of drug screening involve testing thousands of compounds against biological targets, a process that is both labor-intensive and expensive. AI transforms this approach by using computational models to predict the interaction between drug candidates and their targets [151]. Machine learning algorithms can analyze the chemical structures of millions of compounds and predict their binding affinity to specific targets, effectively prioritizing candidates that are more likely to be effective [152,153]. This in silico screening drastically reduces the number of compounds that need to be physically tested, saving time and resources.

AI also facilitates the repurposing of existing drugs for new therapeutic uses [149,154,155]. By analyzing patterns in clinical data and understanding the molecular mechanisms of existing drugs, AI can identify new applications for these compounds. For instance, drugs initially developed for other cancers or diseases may exhibit potential efficacy against HNC, offering a faster and more cost-effective route to developing new treatments [156,157].

Moreover, AI-driven drug discovery is enhanced by its ability to integrate diverse datasets, providing a more comprehensive understanding of drug interactions and disease mechanisms [158,159,160]. This integrative approach combines data from clinical trials, electronic health records, scientific literature, and real-world evidence to uncover hidden connections and insights. By leveraging this wealth of information, AI can generate hypotheses and guide experimental design, further accelerating the discovery process.

### 5.2. Precision Medicine

Precision medicine aims to tailor medical treatments to individual patients based on their genetic, molecular, and clinical profiles [161,162,163,164,165,166,167]. Integrating AI with genetic and molecular data is at the forefront of this approach, especially in the treatment of HNC [73,106,168]. Capabilities of AI in analyzing vast datasets and identifying complex patterns enable the customization of therapies to improve efficacy and minimize adverse effects [166].

The role of AI in precision medicine begins with the comprehensive analysis of genetic data [95,169,170]. By sequencing the genomes of cancer patients, researchers can identify specific mutations and alterations that drive the disease. AI algorithms, particularly machine learning models, can process this genomic data to detect mutations that are associated with cancer progression and treatment resistance. This analysis allows for the identification of genetic biomarkers that can guide the selection of targeted therapies. For example, if a patient’s tumor exhibits a particular mutation that is known to respond well to a specific drug, AI can help ensure that this drug is included in the treatment plan.

Beyond genetic data, AI integrates other molecular information, such as proteomics and metabolomics, to provide a more holistic view of the patient’s disease [171,172]. Proteomics involves studying the entire set of proteins expressed by the genome, while metabolomics focuses on the chemical processes and metabolites within cells. AI can analyze proteomic data to identify protein expression patterns that are unique to cancer cells, revealing potential targets for therapy [95,143,173]. Similarly, by examining metabolic alterations in cancer cells, AI can uncover vulnerabilities that can be exploited with specific drugs. Integrating these diverse data types enhances the precision of treatment customization, as it accounts for multiple aspects of the tumor’s biology.

AI also excels in predicting treatment responses based on genetic and molecular profiles. Machine learning models can be trained on historical patient data, including treatment outcomes and genetic information, to predict how new patients will respond to various therapies. These predictions help oncologists select the most effective treatments for each patient. For instance, if AI analysis indicates that a patient’s molecular profile is similar to that of others who responded well to immunotherapy, this treatment can be prioritized [117]. Conversely, if the profile suggests a high likelihood of resistance to a particular chemotherapy, alternative options can be considered [37,174].

The integration of AI with genetic and molecular data also supports the development of combination therapies [138,153]. AI can analyze data to identify synergistic effects between different drugs, suggesting combinations that might be more effective than single-agent therapies. This approach is particularly valuable in treating cancers that are resistant to conventional treatments. By customizing combination therapies based on a patient’s unique molecular profile, AI helps maximize therapeutic efficacy and minimize the likelihood of resistance.

Furthermore, AI-driven precision medicine extends to monitoring and adjusting treatments in real time [175,176]. The continuous analysis of patient data, such as changes in tumor size, biomarker levels, and side effects, enables AI systems to adapt treatment plans dynamically. For example, if a tumor starts to grow despite ongoing therapy, AI can analyze the new data to recommend adjustments or alternative treatments [107,126]. This real-time adaptability ensures that treatments remain effective throughout the course of the disease.

### 5.3. Surgery

AI is significantly enhancing the field of robotic surgery [177,178], including the treatment of HNC [123,179]. AI-assisted surgical techniques offer increased precision, improved outcomes, and reduced recovery times, marking a substantial advancement over traditional surgical methods.

AI plays a critical role in preoperative planning for HNC surgeries by analyzing patient data to create highly individualized surgical plans [180,181,182]. Utilizing advanced imaging technologies like high-resolution CT and MRI, AI algorithms can generate comprehensive images of the oral cavity and surrounding structures. These algorithms then precisely identify the location, size, and extent of the tumor, along with vital anatomical features such as nerves and blood vessels that must be preserved. This in-depth mapping allows surgeons to design highly accurate surgical approaches, ensuring maximal tumor removal while minimizing the risk of damaging healthy tissues and critical structures.

During surgery, AI-assisted robotic systems can provide surgeons with enhanced capabilities [183,184]. These systems can offer superior dexterity and stability compared to human hands, allowing for more precise movements in the confined and complex spaces of the oral cavity. AI algorithms can assist by providing real-time feedback. For instance, the system can alert the surgeon if they are approaching critical structures or if there are any deviations from the planned path. This real-time assistance helps to reduce the risk of complications and ensures that the tumor is removed with greater accuracy.

Robotic systems equipped with AI can also facilitate minimally invasive surgical techniques [178,184,185], which are associated with several benefits over traditional open surgeries. Minimally invasive procedures typically result in smaller incisions, less blood loss, reduced pain, and faster recovery times. AI enhances these procedures by enabling precise control over the surgical instruments, allowing for delicate and complex maneuvers that would be challenging with conventional techniques. For patients with HNC, this can mean shorter hospital stays and a quicker return to normal activities.

The impact of AI on surgical training and education is also impressive [186,187,188,189,190,191]. AI-based robotic systems can be used to simulate various surgical scenarios, allowing surgeons to practice and refine their skills in a controlled environment. AI can provide immediate feedback and assess performance, highlighting areas for improvement. This enhances the training experience and helps to ensure that surgeons are well prepared for real-life operations.

## 6. Challenges and Limitations

### 6.1. Data Quality and Accessibility

AI holds great promise in advancing the diagnosis and treatment of HNC [33,71,97,192], but its efficacy is heavily dependent on the quality, standardization, and accessibility of data. Several issues, particularly regarding data privacy, standardization, and availability, need to be addressed to harness the full potential of AI in this field [32,51,193].

Data privacy is a major concern when it comes to using patient information for AI applications. Healthcare data are highly sensitive, containing personal, genetic, and medical details that, if mishandled, can lead to serious breaches of patient confidentiality and trust. The integration of AI requires robust data security measures to protect this information from unauthorized access, cyber-attacks, and other forms of misuse. Compliance with regulations such as the Health Insurance Portability and Accountability Act (HIPAA) in the United States [194] and the General Data Protection Regulation (GDPR) in Europe (https://gdpr-info.eu) is essential. These regulations mandate strict guidelines on how patient data should be collected, stored, and shared, ensuring that privacy is maintained. Anonymization and encryption techniques are commonly employed to protect patient identities while allowing data to be used for research and AI training.

The standardization of data is crucial for the successful application of AI in cancer care and treatment [195]. Data collected from different sources, such as hospitals, research institutions, and clinics, often vary in format, quality, and content. This lack of uniformity can hinder the effective analysis and integration of data. Standardized protocols for data collection and reporting ensure that information is consistent and comparable across different datasets. For instance, imaging data must follow specific formats like DICOM (Digital Imaging and Communications in Medicine) to be universally interpretable. Similarly, clinical data should adhere to standardized coding systems such as ICD (International Classification of Diseases) and SNOMED CT (Systematized Nomenclature of Medicine–Clinical Terms). Ensuring that data adhere to these standards facilitates interoperability and enhances the accuracy and reliability of AI models.

The availability of data is another critical issue [47,72,97,196]. AI algorithms require large, diverse datasets to train effectively and make accurate predictions. However, accessing sufficient data can be challenging due to various factors. Many institutions are reluctant to share data due to privacy concerns, proprietary interests, or logistical barriers. Additionally, there might be disparities in data availability, with well-resourced institutions possessing extensive datasets while smaller or less-funded institutions struggle to collect comprehensive data. Collaborative efforts and data-sharing initiatives are essential to overcome these barriers. Establishing centralized databases or data repositories, where institutions can contribute and access pooled data, can significantly enhance data availability. These repositories must ensure compliance with privacy laws and ethical standards while promoting open data sharing.

The quality of data is paramount for the effectiveness of AI models [47,72,97,197]. Poor-quality data, such as incomplete records, errors in data entry, or inconsistent measurements, can lead to inaccurate predictions and unreliable outcomes. Ensuring high data quality involves rigorous data cleaning and validation processes. This includes verifying the accuracy of data entries, correcting errors, filling in missing values, and ensuring that the data are up to date. Regular audits and quality control checks can help maintain data integrity. Additionally, training healthcare personnel on proper data collection and management practices is essential to prevent errors and ensure the reliability of the data.

### 6.2. Algorithm Bias and Fairness

Addressing biases in AI models and ensuring equity in healthcare are critical issues in the application of artificial intelligence, particularly in the treatment of HNC and care [198,199]. Algorithmic bias can lead to disparities in healthcare outcomes, affecting the accuracy and fairness of AI-driven interventions. Ensuring that AI models are fair and unbiased is essential for providing equitable healthcare to all patients.

Algorithmic bias occurs when AI models produce systematically unfair outcomes for certain groups of people. This bias can arise from various sources, including biased training data, biased algorithms, and the subjective decisions made during model development. In the context of HNC treatment, biases in AI models can result in unequal access to accurate diagnoses, effective treatments, and overall healthcare services. For example, if the training data predominantly include patients from a particular demographic group, the AI model might perform well for that group but poorly for others, leading to disparities in treatment outcomes.

One primary source of bias is the data used to train AI models. If the training data is not representative of the entire patient population, the resulting model may not generalize well to underrepresented groups. This issue is particularly relevant in healthcare, where data can be skewed due to various factors such as socioeconomic status, geographic location, and racial or ethnic background. For instance, if a dataset used to train an AI model for HNC diagnosis predominantly consists of data from affluent urban areas, the model may not perform well for patients from rural or low-income areas.

Bias can also stem from the algorithms themselves. Certain algorithms may inadvertently prioritize features that are more prevalent in certain groups, leading to biased outcomes. Additionally, the subjective decisions made during the model development process, such as feature selection and parameter tuning, can introduce biases if not carefully managed.

Mitigating algorithmic bias involves several strategies. First, it is essential to ensure that the training data is diverse and representative of the entire patient population. This can be achieved by including data from a wide range of sources and demographic groups. Collaborating with various healthcare institutions to pool data can help create a more comprehensive and balanced dataset. Additionally, techniques such as data augmentation and synthetic data generation can be used to enhance the diversity of the training data.

Second, bias detection and correction methods should be implemented during the model development process. This includes using metrics to evaluate the model performance across different demographic groups and identifying any disparities. If biases are detected, techniques such as re-weighting, re-sampling, and adversarial debiasing can be employed to mitigate them. These methods can adjust the training process to ensure that the model treats all groups fairly.

Third, transparency and accountability are crucial in addressing algorithmic bias. AI developers should document the entire development process, including the sources of training data, the methods used to address bias, and the model performance across different groups. This transparency allows for external review and validation, ensuring that the model is robust and unbiased. Additionally, involving diverse teams in the development process can help identify and address potential biases from different perspectives.

Ensuring equity in healthcare goes beyond mitigating algorithmic bias. It involves creating AI models that actively promote fair and equitable treatment for all patients. This includes considering social determinants of health, such as socioeconomic status, education, and access to care, when developing AI models. By integrating these factors into the models, AI can help identify and address disparities in healthcare access and outcomes.

Moreover, patient engagement and education are crucial for ensuring equity. Patients should be informed about how AI is being used in their care and the steps taken to ensure fairness and accuracy. This transparency builds trust and encourages patient participation in AI-driven healthcare initiatives.

Policies and regulations also play a vital role in promoting equity. Regulatory bodies should establish guidelines and standards for AI development and deployment in healthcare, ensuring that models are thoroughly tested for fairness and bias before being implemented. Regular audits and reviews of AI systems can help maintain accountability and address any emerging biases.

Addressing biases in AI models and ensuring equity in healthcare are essential for the successful integration of AI in HNC treatment. By employing diverse and representative data, implementing bias detection and correction methods, promoting transparency and accountability, and considering social determinants of health, AI models can be developed to provide fair and equitable healthcare to all patients. These efforts are crucial for harnessing the full potential of AI to improve health outcomes and reduce disparities in HNC care.

### 6.3. Integration into Clinical Practice

Implementing AI tools in real-world clinical settings, particularly for HNC treatment, presents several challenges [32,36,47,74,200,201,202,203,204]. While AI has the potential to revolutionize healthcare by enhancing diagnostic accuracy, personalizing treatments, and improving patient outcomes, the transition from theoretical models to practical applications involves navigating complex issues. These challenges include technological integration, data management, clinical validation, regulatory compliance, and acceptance by healthcare professionals and patients.

One of the primary challenges in integrating AI tools into clinical practice is the technological infrastructure required. Many healthcare facilities may lack the necessary hardware and software to support advanced AI applications. Implementing AI systems often requires significant investments in computational power, secure data storage solutions, and interoperability with existing EHR systems. Ensuring seamless integration with EHRs is particularly crucial, as it allows AI tools to access and analyze patient data effectively. Overcoming these technological barriers involves upgrading current systems and ensuring that new technologies are compatible with a wide range of healthcare platforms.

Before AI tools can be widely adopted in clinical practice, they must undergo rigorous clinical validation to ensure their safety, efficacy, and reliability [205,206,207,208]. This process involves extensive testing in real-world settings to confirm that AI models perform accurately and consistently across diverse patient populations. Clinical validation requires collaboration between AI developers, healthcare providers, and regulatory bodies to design and conduct trials that adequately assess the performance of developed AI tools. The time and resources required for such validation can be substantial, and the process must address potential biases and variations in data to ensure the generalizability of AI solutions.

Regulatory compliance is another significant hurdle in the integration of AI into clinical practice [207,209]. AI tools used in healthcare are subject to regulatory scrutiny to ensure they meet standards for safety and effectiveness. In the United States, the Food and Drug Administration (FDA) oversees the approval of medical devices and software, including AI applications. Navigating the regulatory landscape involves understanding and meeting the requirements for clinical trials, documentation, and reporting. Regulatory agencies are still developing frameworks specific to AI, which adds an element of uncertainty for developers and healthcare providers. Ensuring compliance with evolving regulations is essential for the successful and sustainable implementation of AI tools.

The acceptance of AI tools by healthcare professionals is crucial for their successful integration into clinical practice [210,211]. Healthcare providers may be skeptical of AI’s reliability or concerned about its impact on their clinical autonomy. Building trust in AI systems requires demonstrating their value through evidence-based results and providing training to ensure that professionals can effectively use these tools. Additionally, clear communication about the benefits and limitations of AI is essential to address any concerns and promote adoption.

Patients’ acceptance is equally important [211,212,213,214]. Educating patients about how AI tools can enhance their care, ensuring transparency in how their data are used, and addressing privacy concerns can help build confidence in AI applications. Engaging patients in the implementation process and incorporating their feedback can also improve the design and functionality of AI tools, making them more user-friendly and effective.

Integrating AI tools into the existing clinical workflow without causing significant disruptions is another challenge [215,216,217,218]. AI systems must be designed to complement the work of healthcare providers, enhancing their capabilities rather than adding to their workload. This involves creating intuitive interfaces, minimizing the time required to input and analyze data, and providing actionable insights that seamlessly fit into clinical decision-making processes. Workflow integration requires collaboration between AI developers and healthcare practitioners to ensure that the tools are practical and aligned with the realities of clinical practice.

## 7. Future Directions

Table 3 presents an overview of the diverse applications of AI in HNC, detailing specific examples and the advantages each application offers. Similarly, Table 4 summarizes various AI techniques utilized in HNC research, outlining their specific applications and the benefits they provide. This comparative analysis aids readers in grasping the array of AI tools available and their contributions to advancements in HNC detection, diagnosis, and treatment.

Looking ahead, several promising avenues for research and clinical applications of AI in HNC warrant exploration, including the following.

### 7.1. Potential Solutions to HNC

#### 7.1.1. Diverse Mutational Landscape

The diverse mutational landscape observed in HNC refers to the wide array of genetic alterations and mutations that can occur within these tumors [3,226,227]. This variability is influenced by factors such as the anatomical site of the cancer, exposure to carcinogens like tobacco and alcohol, and infection with viruses like HPV (human papillomavirus).

Key mutations might include alterations in genes like TP53, which is commonly mutated in HNC, as well as mutations in genes involved in cell cycle regulation, DNA repair, and signaling pathways such as EGFR, PIK3CA, and Notch1 [228,229]. This complexity results in a heterogeneous disease where different patients may have vastly different genetic profiles, even within the same subtype of HNC.

Such a diverse mutational landscape presents both challenges and opportunities for AI applications. AI can be utilized to analyze large datasets of genetic information, identifying patterns and correlations that might not be apparent through traditional methods. For instance, AI algorithms can help predict which mutations are likely to drive cancer progression, or which patients might respond better to certain treatments based on their unique genetic profiles. Additionally, AI can assist in stratifying patients for clinical trials, personalizing treatment plans, and even discovering new therapeutic targets by exploring the complex interplay of mutations within the tumor microenvironment.

By leveraging AI to interpret the diverse mutational landscape of HNC, there is potential to significantly advance precision medicine, tailoring interventions to the specific genetic makeup of each patient’s cancer.

#### 7.1.2. Surgical Planning and Management

AI-based solutions can offer significant potential to enhance the complex planning and implementation of surgical management in HNC. These technologies address several key challenges, including functional preservation [230], precise tumor resection [231], and the effective use of grafts and reconstruction techniques [232,233,234].

In the preoperative phase, AI can assist by creating detailed three-dimensional models of the tumor and surrounding anatomy from imaging data. These models allow surgeons to visualize the precise location and extent of the cancer, facilitating careful planning of the surgical approach. This reduces the risk of damaging critical structures like nerves, blood vessels, and muscles that are essential for functions such as speech and swallowing. Additionally, AI-driven platforms can simulate various surgical scenarios, enabling surgeons to anticipate potential complications and optimize their strategies accordingly. By predicting the outcomes of different approaches, AI helps in selecting the most effective and minimally invasive surgical plan.

During surgery, AI can provide real-time guidance through advanced image analysis. For instance, it can analyze intraoperative scans such as CT or MRI to guide the surgeon with exceptional precision. This ensures accurate tumor resection while preserving as much healthy tissue as possible. Furthermore, AI can power augmented reality systems that overlay critical information onto the surgeon’s field of view. This technology enhances the surgeon’s ability to navigate complex anatomy and avoid functional impairments by highlighting important anatomical landmarks and suggesting optimal cutting paths.

AI can also play a crucial role in reconstruction and graft planning. It assists in designing personalized grafts tailored to the patient’s unique anatomy by analyzing the defect created by tumor resection. This results in better functional and aesthetic outcomes. AI models can also predict how different graft materials will integrate with the patient’s existing tissue, helping to choose the most compatible options. This reduces the likelihood of graft failure and promotes better healing and functionality.

In the postoperative phase, AI can contribute by predicting recovery outcomes and guiding rehabilitation. By analyzing preoperative and intraoperative data, AI can identify patients at higher risk of functional impairments, enabling early interventions that improve recovery. Additionally, AI algorithms can predict long-term functional outcomes based on the surgical approach and extent of tissue resection, providing valuable insights that help refine surgical techniques and enhance the quality of life for HNC patients.

#### 7.1.3. Radiotherapy and Adaptive Radiotherapy

The implementation of radiotherapy in the head and neck region is particularly laborious and challenging due to the complex anatomy, the proximity of critical structures, and the need for precise targeting to minimize damage to healthy tissue [120,235]. AI-based solutions have the potential to make this process more efficient and effective, addressing the unique challenges associated with radiotherapy for HNC [236].

One significant advantage of AI in radiotherapy planning is its ability to automate and optimize the delineation of target areas and critical organs [237,238]. Traditionally, this task is highly manual and time-consuming, requiring meticulous contouring by radiation oncologists. AI can streamline this process by analyzing imaging data, such as CT or MRI scans, to accurately delineate tumor boundaries and nearby organs at risk. This automation not only saves time but also enhances precision, reducing the likelihood of human error and ensuring that the radiation dose is concentrated on the tumor while sparing healthy tissue.

Furthermore, AI can optimize treatment planning by predicting the optimal radiation dose distribution [120]. By analyzing large datasets from previous patients, AI can identify patterns and correlations that inform the best treatment strategies for individual cases. This includes adjusting radiation doses to account for variations in tumor size, shape, and location, as well as the presence of any critical structures nearby. AI-driven treatment planning can also adapt to changes in tumor size or position during the course of treatment, ensuring that the therapy remains effective throughout its duration.

AI has the potential to enhance patient-specific radiotherapy by predicting treatment outcomes and side effects [238,239]. By analyzing data from similar cases, AI can forecast how a patient is likely to respond to a particular radiotherapy regimen. This predictive capability allows for the customization of treatment plans to maximize effectiveness and minimize adverse effects, particularly in the delicate and complex head and neck region. Additionally, AI can help in identifying patients who may benefit from advanced techniques like intensity-modulated radiotherapy or proton therapy, which offer greater precision in targeting tumors.

Despite significant advancements in radiotherapy for head and neck squamous cell carcinoma, such as advanced intensity modulation and daily image-guided techniques, these innovations still fall short in addressing the inherent structural and spatial changes that can occur during the course of treatment. Numerous studies have documented reductions in the volumes of the primary tumor, lymph nodes, and parotid glands throughout treatment, which can lead to unintended changes in dose distribution. These dosimetric alterations may impact both the side effect profile and the overall effectiveness of the therapy. Adaptive radiotherapy has emerged as an exciting approach specifically designed to dynamically adjust treatment in response to these anatomical changes, reducing excessive radiation exposure to critical organs [240,241].

During the actual delivery of adaptive radiotherapy, AI can provide real-time monitoring and adjustments [242]. Advanced imaging techniques, combined with AI, allow for continuous tracking of the tumor and surrounding tissues during treatment sessions. This real-time feedback enables precise adjustments to the radiation beam, compensating for any patient movement or changes in anatomy that could otherwise compromise the accuracy of treatment. As a result, the radiation dose can be delivered more accurately, improving the chances of eradicating the tumor while minimizing side effects.

#### 7.1.4. Multiple Simultaneous Primary Tumors

In HNC, the occurrence of second or synchronous primary tumors is a common phenomenon [243,244,245]. These additional tumors can emerge either simultaneously with the initial cancer or as a subsequent primary cancer in a different location within the head and neck region. Managing these multiple tumors presents unique challenges, as each tumor may require distinct treatment strategies, and the presence of multiple cancers complicates the overall management plan.

Given the complexity of detecting and treating multiple primary tumors, this area is particularly well-suited for AI applications. AI can be employed to enhance the detection and differentiation of these tumors through advanced imaging analysis with deep learning, ensuring that each tumor is accurately identified and appropriately addressed. Additionally, AI could help in the development of personalized treatment plans that account for the presence of multiple tumors, optimizing the approach to minimize the impact on healthy tissue while effectively targeting all cancerous growths.

Furthermore, AI-driven predictive models could be utilized to assess the likelihood of developing second primary tumors, enabling earlier intervention and tailored surveillance strategies. By integrating AI into the management of multiple primary tumors in HNC, healthcare providers can improve both the accuracy of diagnosis and the effectiveness of treatment, ultimately leading to better outcomes for patients facing this complex condition.

### 7.2. Innovations on the Horizon

Emerging AI technologies hold immense potential to further transform the field of HNC diagnosis, treatment, and patient care. These advancements promise to enhance the precision, efficiency, and personalization of healthcare delivery, ultimately improving patient outcomes. Key innovations include advanced machine learning algorithms, explainable AI, AI-powered robotics, and the integration of multimodal data.

#### 7.2.1. Advanced Machine Learning Algorithms

New machine learning algorithms are being developed to improve the accuracy and robustness of AI models used in HNC detection and treatment. Deep learning techniques, such as CNNs, RNNs, and vision transformers, are becoming more sophisticated, enabling them to analyze complex medical images and patient data with higher precision. These algorithms can identify subtle patterns and anomalies that may be missed by human observers, leading to earlier and more accurate diagnoses. Additionally, ensemble learning, which combines multiple models to improve prediction accuracy, is gaining traction. These advanced algorithms can provide more reliable prognostic assessments, aiding in the development of personalized treatment plans.

#### 7.2.2. Explainable Machine Intelligence

Explainable AI (XAI) is an emerging field focused on making AI models more transparent and interpretable (https://cloud.google.com/explainable-ai (accessed on 7 May 2024)). In healthcare, the ability to understand and trust AI decisions is crucial for both practitioners and patients. XAI techniques aim to provide clear explanations of how AI models arrive at their conclusions, enabling clinicians to validate and trust the recommendations. For instance, in HNC diagnosis, XAI can help elucidate why a particular lesion was classified as malignant, highlighting the specific features and patterns that influenced the decision. This transparency fosters trust and facilitates the integration of AI into clinical practice, as healthcare professionals can confidently rely on AI insights while maintaining control over patient care decisions.

#### 7.2.3. Integration of Multimodal Data

The integration of multimodal data is an emerging trend that leverages AI to combine various types of data for a comprehensive understanding of HNC [38,106,246]. This approach involves integrating data from medical imaging, genomics, proteomics, and clinical records to create a holistic view of the patient’s condition. AI algorithms can analyze these diverse data to uncover correlations and insights that may not be evident when considering each data type in isolation. For example, combining imaging data with genetic information can improve the accuracy of cancer staging and identify specific mutations that may influence treatment response. This multimodal analysis enables more precise and personalized treatment plans, leading to better patient outcomes.

#### 7.2.4. AI in Drug Discovery

AI is revolutionizing the field of drug discovery for HNC treatment. Advanced algorithms can analyze vast datasets of chemical compounds and biological interactions to identify potential new drugs and therapeutic targets. Machine learning models can predict the efficacy and safety of these compounds, significantly accelerating the drug development process. AI-driven drug discovery holds the promise of identifying novel treatments that are more effective and have fewer side effects, offering new hope for patients with HNC. Additionally, AI can optimize existing treatment regimens by identifying the best combinations and dosages based on individual patient profiles.

#### 7.2.5. Real-Time Monitoring and Decision Support

Emerging AI technologies are enhancing real-time monitoring and decision support systems in HNC care [137]. Wearable devices and sensors can continuously collect data on patients’ vital signs, activity levels, and other health metrics. AI algorithms can analyze these datasets in real time to detect early signs of complications, monitor treatment response, and provide timely alerts to healthcare providers. These systems will enable proactive and personalized care, allowing clinicians to intervene promptly when necessary. Furthermore, AI-powered decision support tools can assist healthcare professionals by providing evidence-based recommendations and predicting the likely outcomes of different treatment options, thereby enhancing clinical decision-making.

#### 7.2.6. Telemedicine and Remote Care

The integration of AI with telemedicine platforms is another promising innovation for early diagnosis of HNC and care, proving most advantageous to underserved populations [74,247,248]. AI-powered tools can enhance remote consultations by analyzing patient data and providing diagnostic support to clinicians. For patients with HNC, telemedicine can facilitate regular follow-ups, remote monitoring, and timely interventions, reducing the need for frequent hospital visits. AI can also assist in triaging patients, identifying those who require urgent in-person care versus those who can be managed remotely. This approach can improve access to care, especially for patients in underserved or rural areas, and ensures continuous and comprehensive management of HNC.

### 7.3. Interdisciplinary Collaboration

The integration of AI into HNC treatment and research highlights the critical importance of interdisciplinary collaboration [249,250]. Effective implementation of AI technologies in healthcare requires the combined expertise of AI experts, clinicians, and researchers. This collaborative approach is essential for developing AI tools that are both clinically relevant and scientifically robust, ensuring that these innovations translate into meaningful improvements in patient care.

Clinicians play a pivotal role in ensuring that AI tools address real-world challenges and are tailored to the practical needs of patient care. Their insights into the day-to-day complexities of diagnosing and treating HNC are invaluable for guiding the development of AI applications. By working closely with AI experts, clinicians can provide feedback on the specific clinical problems that need to be addressed, such as the complexity of interpreting imaging data or the challenges of integrating AI recommendations into existing workflows. This collaboration helps ensure that AI models are designed to solve pressing clinical issues and are aligned with the realities of patient care.

Interdisciplinary collaboration is also crucial for addressing ethical and practical concerns associated with AI in healthcare. Clinicians can help identify potential ethical issues related to patient consent, data privacy, and the equitable use of AI technologies. They ensure that AI tools are implemented in ways that respect patient autonomy and comply with regulatory requirements. Researchers and AI experts can contribute by developing methods to mitigate biases, ensure data security, and maintain transparency in AI decision-making processes. Together, these professionals work to create AI systems that are ethical, secure, and aligned with the principles of patient-centered care.

Effective collaboration among AI experts, clinicians, and researchers is essential for education and training in the use of AI tools. Clinicians need to be trained in how to use AI systems effectively and understand their limitations and strengths. Researchers and AI experts can develop educational programs and training modules that bridge the gap between technological innovations and clinical practice. This training ensures that healthcare professionals are well-prepared to integrate AI tools into their practice, enhancing their ability to provide high-quality care and make informed decisions.

The dynamic nature of both AI technology and clinical practice necessitates ongoing collaboration for continuous improvement. As AI tools evolve and new research findings emerge, clinicians, researchers, and AI experts must work together to update and refine these tools. Feedback from clinical practice informs further development, while advancements in research and technology drive innovations that can be incorporated into clinical workflows. This iterative process ensures that AI tools remain relevant, effective, and aligned with the latest developments in HNC treatment.

Fostering a culture of collaboration is essential for maximizing the benefits of AI in HNC care. This involves creating platforms for regular communication and interaction among AI experts, clinicians, and researchers. Collaborative projects, joint research initiatives, and multidisciplinary conferences can facilitate knowledge sharing and problem-solving. Building strong partnerships across disciplines helps to overcome challenges, leverage diverse expertise, and drive innovation in HNC treatment.

## 8. Study Limitations

The current study may have the following limitations:

1. Scope of coverage: the review might focus primarily on recent advances in AI without fully addressing earlier foundational work, potentially limiting the understanding of the evolution of AI applications in HNC.
2. Selection bias: the review could be biased towards studies that demonstrate positive results, neglecting those with negative or inconclusive outcomes, which is crucial for a balanced understanding of the effectiveness of AI.
3. Heterogeneity in studies: the review includes studies with varying methodologies, datasets, and patient populations, making it difficult to compare outcomes or draw definitive conclusions about the general applicability of AI techniques.
4. Lack of practical implementation insights: the review might focus more on research aspects rather than practical challenges and considerations for implementing AI tools in clinical settings, such as integration into existing workflows and real-world validation.
5. Clinical validation: this review could be limited in its discussion of the clinical validation and real-world efficacy of AI applications, as many studies may be preliminary or based on small sample sizes, requiring further validation in broader clinical contexts.

These limitations suggest that, while the review provides valuable insights into the potential of AI in HNC, further research and consideration are needed to address these gaps and ensure the successful translation of AI innovations into clinical practice.

## 9. Conclusions

The integration of AI into the domain of HNC presents transformative opportunities that promise to significantly advance the field. This review has highlighted several key findings, including the diverse AI techniques currently in use, the challenges and opportunities associated with their implementation, and potential innovations.

The ability of AI to enhance early detection and screening of HNC through advanced imaging techniques and automated lesion detection has already shown promising results. Deep learning approaches, such as CNNs, RNNs, and vision transformers, are pushing the boundaries of accuracy and efficiency in diagnostic processes. Furthermore, NLP and integration with EHRs and genomics are paving the way for more personalized treatment plans and comprehensive patient management strategies.

However, the successful integration of AI into clinical practice is not without challenges. Issues related to data quality and accessibility, algorithmic bias, and the need for interdisciplinary collaboration are critical to address. Ensuring that AI tools are developed and implemented in a manner that is ethical, transparent, and equitable is essential for gaining trust from both clinicians and patients. Moreover, the continuous need for validation and compliance with regulatory standards underscores the importance of robust and well-documented development processes.

The implications for clinical practice and research are profound. Clinicians can expect more accurate diagnostic tools and personalized treatment options, leading to improved patient outcomes and more efficient healthcare delivery. Researchers will benefit from enhanced capabilities in data analysis, biomarker discovery, and drug development, contributing to more targeted and effective therapies. The collaborative efforts between AI experts, clinicians, and researchers will be pivotal in refining AI applications and ensuring they meet the practical needs of patient care.

Looking to the future, AI holds immense potential to further revolutionize the field of HNC. Emerging technologies, such as advanced machine learning algorithms, XAI, and AI-powered robotics, promise to drive significant advancements. The integration of multimodal data and real-time monitoring systems will provide deeper insights and more tailored approaches to treatment. As these innovations continue to evolve, the synergy between technological advancements and clinical expertise will be crucial in shaping the future landscape of HNC care.

In summary, the future of AI in HNC is one of exciting possibilities. By addressing current challenges and fostering interdisciplinary collaboration, the full potential of AI can be harnessed to enhance diagnostic accuracy, personalize treatments, and ultimately improve patient outcomes. The journey toward integrating AI into HNC care is ongoing, but with continued research, innovation, and collaboration, the promise of AI to transform HNC treatment and management is within reach.

## Figures and Tables

**Table 1:** Demographic and clinical data of patients with oropharynx cancer [30]. Explanation notes: HPV denotes human papillomavirus, BOT denotes the base of the tongue, the T category refers to the description of the original (primary) tumor based on the American Joint Committee on Cancer (AJCC) and Union for International Cancer Control (UICC) cancer staging system. The N category describes whether or not the cancer has reached nearby lymph nodes, based on the AJCC and UICC cancer staging system. Pathological grade indicates the grade of tumor differentiation, where NA = not assessable. Smoking pack-years provide an equivalent numerical value of lifetime tobacco exposure, where one pack-year equals twenty cigarettes smoked every day for one year.

	Patient 1	Patient 2	Patient 3	Patient 4	Patient 5	Patient 6
HPV/p16 status	positive	negative	positive	negative	positive	positive
Gender	male	female	female	male	male	female
Age	58	78	56	59	55	47
Race	white	white	white	black	hispanic	asian
Tumor side	left	right	right	right	right	left
Tumor subsite	tonsil	BOT	BOT	BOT	BOT	BOT
T category	2	3	2	4	2	1
N category	0	0	2b	1	2a	2a
AJCC cancer stage	II	III	IV	III	IV	IV
Pathological grade	III	II	III	NA	III	II–III
Smoking status	former	former	never	current	never	never
Smoking pack-years	5	70	0	66	0	0

**Table 2:** Demographic and clinical data of patients with oral squamous cell carcinoma (OSCC) and leukoplakia (LP) [31]. Explanation notes: age group (years of age): 1 = 41–60 and 2 = over 60, Y = yes, N = no, NI = not informed, FOM = floor of mouth, and BM = buccal mucosa.

	Patient 1	Patient 2	Patient 3	Patient 4	Patient 5	Patient 6
Gender	male	male	male	male	male	male
Age group	1	1	2	2	1	1
Race	white	white	black	white	NI	white
Tobacco use	Y	N	N	former	NI	Y
Alcohol consumption	former	Y	Y	former	NI	Y
Localization	tongue	FOM	BM	gingiva	palate	tongue
Diagnosis	OSCC	OSCC	LP (mild)	OSCC	LP (severe)	OSCC

**Table 3:** Applications of AI in head and neck cancer.

Application	Examples	Benefits
Early Detection [33,71,72,73,74,75,76,77,78,79,80,81,82,83,84,85,86]	Histopathological analysis: digitized slides of tissue samples are fed into an AI system. The AI can highlight areas of concern, rank the likelihood of malignancy, and even provide a second opinion. Imaging techniques: an AI system can analyze image scans to identify tiny nodules or irregular tissue structures that may suggest the presence of cancer. It can also compare current scans with previous ones to track tumor progression.	Improved accuracy in detecting early-stage lesions (quantitative assessments, reduction of false negatives), enhanced visualization of suspicious areas.
Diagnosis [72,74]	Automated lesion detection: an AI system can identify potential lesions, and generate a report highlighting suspicious areas. Classification algorithms: for instance, a lesion found in the oral cavity might be classified as high-risk for squamous cell carcinoma based on its irregular borders and rapid growth rate as identified by the AI. The system might also provide a probability score indicating the likelihood of malignancy, aiding in deciding whether to proceed with a biopsy or other diagnostic tests.	Standardized diagnostic criteria (uniform diagnostics), reduction in diagnostic variability (consistent decision-making, quality control, and second opinions).
Treatment Planning [47,75,112,113,114,115,116,117,118,119,120,121,122,123,124,125,126,127]	Personalized medicine: after a diagnosis of HNC, the patient’s comprehensive data—including medical history, imaging results, and genetic information—are fed into an AI system. The AI analyzes the data to predict the patient’s response to different treatment modalities. Genomics integration: AI can match specific genetic mutations identified in a patient’s tumor with drugs known to target those mutations.	Tailored treatment regimens (optimal therapy selection), better patient outcomes based on individual profiles (adaptive treatment strategies).
Monitoring and Surveillance [128,129,130,131,132,133,134,135,136,137,138,139,140,141,142]	Real-time monitoring: AI can analyze data of vital signs provided by a wearable device and alerts if it detects signs of complications of a patient recovering from HNC surgery. Recurrence detection: an AI system can compare present scans with previous images, looking for any changes in tissue density or new growths. Patient compliance tracking: a patient undergoing chemotherapy for HNC might use a mobile app that sends reminders to take their medication. The AI within the app can monitor the patient’s adherence and flag any missed doses.	Early identification of recurrence, improved patient follow-up, enhanced compliance with treatment protocols.
Drug Discovery [143,144,145,146,147,148,149,150,151,152,153,154,155,156,157,158,159,160]	Identification of therapeutic targets: AI can analyze genomic data to identify mutations like PIK3CA in HNC, leading to the development of targeted treatments such as PI3K inhibitors. Drug screening and repurposing: AI can rapidly screen millions of chemical compounds to identify those most likely to bind to a new head and neck cancer target, streamlining the drug discovery process by prioritizing the most promising candidates for lab testing.	Accelerated drug development (reduced time to market, cost efficiency), identification of novel therapeutic compounds.
Robotics and Surgery [123,179,180,181,182,183,184,185,186,187,188,189,190,191]	AI-assisted surgery: an AI-assisted robotic system can integrate preoperative imaging and analyze real-time data to guide the surgeon, reducing complications and enhancing precision. Precision surgery techniques: an AI-driven laser system can precisely remove tumors near the vocal cords by adjusting in real time to preserve the vocal cords while effectively excising the cancer, improving outcomes and preserving the patient’s voice.	Increased surgical precision (enhanced tumor removal, preservation of critical structures), reduced intraoperative risks, faster recovery times.

**Table 4:** AI techniques and their applications in head and neck cancer.

AI Technique	Applications	Description and Benefits
Supervised Learning [36,37,38]	Image classification, predictive modeling: classifying imaging scans to detect head and neck tumors with high accuracy, while predictive modeling can forecast patient outcomes, such as the likelihood of tumor recurrence, based on clinical and imaging data, helping to tailor treatment strategies.	Utilizes labeled data to train models for accurate diagnosis and prognosis prediction, improving early detection and personalized treatment plans
Unsupervised Learning [38,39,40]	Biomarker discovery, patient clustering: analyzing genomic data and to cluster patients into subgroups based on genetic profiles, leading to more targeted therapies.	Analyzes unlabeled data to identify patterns and correlations, aiding in the discovery of novel biomarkers and understanding patient subgroups
Reinforcement Learning [41,42,43,44,45]	Treatment optimization, robotic surgery: optimizing HNC treatments by adjusting radiation doses based on patient response and enhances robotic surgery precision through real-time feedback.	Uses feedback loops to optimize treatment strategies and enhance precision in surgical procedures, resulting in better patient outcomes and reduced errors
Deep Learning [47,48,49,50,51,52,53,54,55,56,57]	Histopathological image analysis, radiographic image enhancement: analyzing histopathological images, identifying subtle cellular changes for precise diagnosis, and to enhance radiographic images, improving tumor visibility and aiding in more accurate treatment planning.	Employs neural networks to analyze complex medical images, leading to improved detection and diagnosis accuracy
Natural Language Processing (NLP) [63,64,65,66,67,68,69,70]	Literature mining, patient data analysis: identifying new biomarkers or treatment strategies for HNC, while also reviewing patient records to detect patterns and predict responses to therapies, thereby refining treatment protocols.	Extracts and analyzes information from medical literature and patient records, facilitating better decision-making and research insights
Explainable AI (XAI) [219,220,221,222]	Diagnostic decision support, treatment planning transparency: helping oncologists understand why an AI model recommends a particular diagnosis or treatment plan for HNC by offering clear explanations of the underlying data and decision-making process, thereby increasing trust and confidence in the AI’s recommendations.	Enhances the interpretability of AI models, providing clinicians with understandable insights and supporting transparent decision-making processes
Vision Transformer Networks [49,58,223,224,225]	Medical image classification, lesion detection: analyzing imaging scans of HNC patients to accurately classify tumors and detect subtle lesions, enhancing early diagnosis and treatment planning.	Utilizes advanced transformer models for image analysis, offering superior accuracy in detecting and classifying lesions
